# Facilitators and Barriers to Uptake of Genetic and Cascade Testing in Familial Hypercholesterolemia: a Systematic Review

**DOI:** 10.1007/s12529-025-10357-y

**Published:** 2025-04-08

**Authors:** Chaitanyasre Lenin, Phoebe X. H. Lim, Ashna Nastar, Tavintharan Subramaniam, Sharon Pek, Magdalena Daccord, Elsie Evans, Emma Print, Frederick H. F. Chan, Konstadina Griva

**Affiliations:** 1 Lee Kong Chian School of Medicine, Nanyang Technological University, Singapore, Singapore; 2 Division of Endocrinology, Alexandra Hospital, Singapore, Singapore; 3 Diabetes Centre Admiralty Medical Centre, Division of Endocrinology, Department of Medicine, Khoo Teck Puat Hospital, Singapore, Singapore; 4 Clinical Research Unit, Khoo Teck Puat Hospital, Singapore, Singapore; 5 FH Europe Foundation, Amsterdam, The Netherlands; 6 Lee Kong Chian School of Medicine, Nanyang Technological University, Level 18, Clinical Sciences Building, 11 Mandalay Road, Singapore, 308232 Singapore

**Keywords:** Genetic testing, FH, Cascade testing, Barriers, Facilitators, Cardiovascular disease, Precision medicine, Public health

## Abstract

**Background:**

Familial hypercholesterolemia (FH) is an underdiagnosed autosomal dominant genetic disorder that confers high but preventable risk for premature adverse cardiovascular events. Timely diagnosis is limited by low uptake of genetic testing (GT) and cascade testing (CT). This systematic review identifies barriers and facilitators for uptake of GT and CT in FH.

**Method:**

Following PRISMA guidelines, seven databases were searched for studies on GT/CT in FH. Data reporting standards for qualitative studies were evaluated with COREQ and thematic synthesis was conducted. Of the 387 studies identified, 15 were included (qualitative *N* = 9, quantitative *N* = 6). These involved 272,954 respondents (qualitative *n* = 243, quantitative *n* = 272,711). COREQ scores ranged from 11 to 21 out of 32.

**Results:**

Synthesis of qualitative data indicated family history of illness, being well informed, and value of GT as key facilitators of GT. Financial concerns, suboptimal clinical care, and no/low value of GT were identified as barriers. Facilitators of CT included responsibility to family, healthcare providers’ support for CT, and gains of CT, while barriers included disconnect from family, emotional costs, and no value knowing FH status.

Quantitative studies reflect emotional distress avoidance, limited opportunity for family disclosure to invite, lack of knowledge, low communication efficacy, and difficulties accessing testing services as predictors impacting CT.

**Conclusion:**

Beyond knowledge, perceptions about testing—especially perceived value of testing—emerged to be significantly affecting decisions for GT/CT. Disconnect from family is a maior predictor in CT, reducing the likelihood of probands extending an invitation to their family in support of CT. Future interventions should address barriers and facilitators at interpersonal, clinical and systemic levels to improve FH GT/CT uptake. Additionally, further research in diverse cultural contexts is required to bridge gaps in GT/CT services. Interventions should especially prioritize risk perception education and the development of health communication tools to supplement strong clinical guidance, driving a more patient-centered approach in decisions relating to GT/CT.

**Supplementary Information:**

The online version contains supplementary material available at 10.1007/s12529-025-10357-y.

## Introduction

Familial hypercholesterolemia (FH) is a common genetic autosomal dominant condition involving pathogenic variants in the *LDLR*, *APOB*, and *PCSK9* genes that affects 1 in 311 people globally [1]. FH is recognized as a global public health burden with serious health risks ranging from atherosclerosis to acute myocardial infarction (MI) and sudden cardiac death in undiagnosed individuals, warranting urgent action as per public health guidelines [2, 3]. Characterized by elevated levels of low-density lipoprotein cholesterol (LDL-C), FH results in increased risk of premature atherosclerotic cardiovascular disease [4]. A recent meta-analysis concluded that FH pathogenic variants increase the risk of myocardial infarction by sixfold compared to the risk among those with no FH variant [5]. However, FH diagnosis clinically may not be accurate as physical signs of cholesterol deposits such as xanthomas and corneal arcus only become visible after the onset of coronary heart disease (CHD) [1, 4].

The advances in the Human Genome Program and resulting sequencing technologies are transforming healthcare both in terms of early detection and timely prevention and treatment of a range of conditions including FH. Genetic testing—having revolutionized the diagnosis of other hereditary conditions—is currently the most sensitive technology available in identifying specific genetic mutations responsible for FH [5, 6]. Early diagnosis coupled with early cholesterol lowering has been proven to lower the risk of premature atherosclerotic disease resulting from FH through increasing uptake of lipid-lowering therapy and subsequent attainment of LDL-C targets [7, 8]. These treatments show promise in improving clinical prognosis compared to those with undiagnosed FH [8]. Retrospective cohort studies note that at age 18, life expectancy for those with recorded diagnosis of FH is 16 years longer than those with undiagnosed FH, attributable to risk mitigation via timely pharmacological and lifestyle interventions [8].

However, diagnosis of FH currently continues to be made late, on average, after 40 years, with only 2% made before 18 years, highlighting the urgent need to implement early screening programs globally [9]. Uptake of genetic testing in at-risk relatives is a key limitation in achieving timely diagnosis of FH, with only 4–12% of uptake reported in US families and limited recent data in other contexts [10]. As cardiovascular deaths continue to rise globally, there remains the need to address the low genetic testing uptake for FH, strengthening preventative cardiovascular care. To address this gap, we conducted an in-depth mixed methods systematic review to identify barriers and facilitators underlying genetic testing (GT) and cascade testing (CT) solely for FH.

## Methods

This systematic review was registered in the PROSPERO database (ID: CRD42024521708) and is reported in accordance with Preferred Reporting Items for Systematic Reviews and Meta-analyses (PRISMA) guidelines [11].

### Eligibility/Inclusion Criteria

Studies were considered eligible for the review if they were:

1. Qualitative studies regarding participants’ perspectives on GT services for FH, or reasons for or against undergoing GT/CT for FH. Mixed method studies to be included if the qualitative data were reported separately.
2. Qualitative or mixed method studies that reported data on provider’s perspectives pertaining to individuals’ psychosocial barriers/motivators to the uptake of GT/CT for FH.
3. Quantitative studies on psychosocial barriers/motivators to the uptake of GT/CT for FH.

Studies were excluded if not published in English; if they were case reports, case studies, conference abstracts, or editorials; and if perspectives could not be clearly attributed to a subgroup (i.e., healthcare providers, probands, or family members). Studies involving pediatric assent were also excluded. Since this review deals with delineating CT and GT barriers and facilitators respectively, studies without clearly distinguishable perspectives were excluded to ensure an accurate representation of barriers and facilitators upon data analysis.

### Information Sources and Search Strategy

We searched eight electronic databases: Medline (Ovid), Embase (Ovid), PubMed, CINAHL, PsycINFO, Web of Science, Scopus, and Cochrane Library, for English-language studies from inception through 24 October 2023. We combined FH terms with the following search terms: “genetic testing” and “barrier”, “facilitator”, “perception”, “challenge”, “uptake or understanding”, “experience”, “preference”, or “intention” (see Supplementary Material for detailed search strategy).

**Fig. 1:**
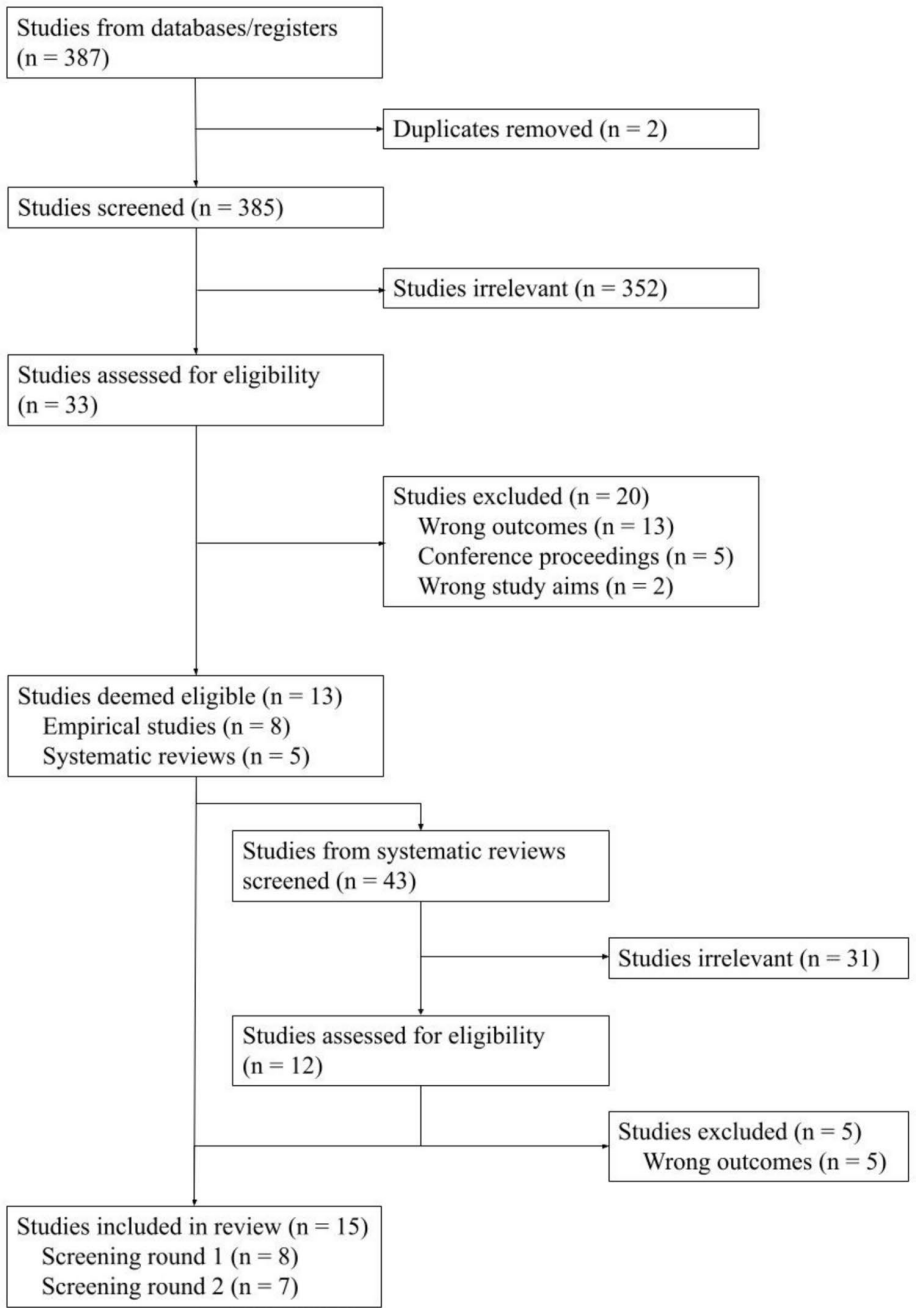
PRISMA flowchart

Reference mining was conducted for all included studies. The flow of procedures can be found in the PRISMA flowchart (Fig. 1).

### Selection Process

Search results were imported into Covidence and two independent reviewers (CL and PL) screened titles and abstracts of retrieved citations. Citations judged as potentially eligible by one or both reviewers were obtained as full text. The full-text publications were then screened against the specified inclusion criteria followed by data abstraction by both raters. Disagreements between the two reviewers were resolved through discussion with a third reviewer (KG). The flow of citations throughout this process was documented in an electronic database, and reasons for exclusion of full-text publications were recorded.

### Data Extraction

The following primary data were extracted: study authors, publication year, study aims, barriers, and facilitators. Additionally, country, setting, years of data collection, study design, sample size, type of participants, and type of test were extracted and are presented as secondary data in a separate table (Table 1).

**Table 1:** Characteristics of studies

Study ID	Country	Setting	Years of data collection	Study design	Sample size	Type of participant	Type of test	COREQ score
Allen et al. (2022)	South Carolina, United States	10 clinics	2021–2022	Qualitative	20	Unknown	Indistinguishable	14
Benson et al. (2016)	United States	2 national databases	2014	Quantitative	761	Unknown	CT	-
Campbell et al. (2017)	Minnesota, United States	State-wide	2016	Quantitative	971	General public	GT and CT	-
Hallowell et al. (2011)	Scotland, United Kingdom	2 clinics, 1 hospital	2010	Qualitative	38	Index patients	CT	15
Hardcastle et al. (2015)	Perth, Australia	1 clinic	Unknown	Qualitative	18 (9 index, 9 relatives)	Index patients and family	GT and CT	11
Jenkins et al. (2013)	Scotland, United Kingdom	2 clinics 1 hospital	2010	Qualitative	38	Index patients	Indistinguishable	15
Schmidlen et al. (2022)	United States	1 forum	2017–2021	Quantitative	270,715	Index and current patient	GT and CT	-
Silva et al. (2022)	United Kingdom	14 clinics and hospitals	Unknown	Qualitative	41 (17 HCP, 24 patients)	Index and current patients Healthcare providers	Indistinguishable	16
Van den Nieuwenhoff et al. (2007)	Netherlands	Nationwide	2003	Qualitative	20	Index patients	CT	21
Van El et al. (2018)	Netherlands	3 states	Unknown	Qualitative	6	Patient organization representative Clinical coordinators Relevant HCPs	CT	17
Wand et al. (2020)	United States	2 forums 5 clinics Database	Unknown	Quantitative	53	FH patient	GT	-
Weiner (2010)	United Kingdom	1 clinic	2004	Qualitative	31	FH patient	GT and CT	16
Weiner & Durrington (2008)	United Kingdom	1 clinic	2004	Qualitative	31	FH patient	GT and CT	13
Wurtmann et al. (2018)	Minnesota, United States	1 clinic	2016	Quantitative	38	Family	GT and CT	-
Zimmerman et al. (2019)	Minnesota, United States	State-wide database	Unknown	Quantitative	173	HCPs	Indistinguishable	-

### Data Synthesis

Thematic analysis was conducted for qualitative studies [12]. Coding was conducted by CL and PL, with a third party involved to resolve disagreements (KG). Refinement of the coding frame and analysis was iterative; codes were identified or merged reading the result sections of identified studies. Resulting themes were reviewed and discussed by all three researchers (CL, PL, and KG), and triangulated by patient and public involvement (PPI) from the FH Europe Foundation to ensure appropriate representation of patient and/or public perspectives on this research to enhance its quality. A review protocol was prepared for this study and registered under the PROSPERO system (ID: CRD42024521708).

Regarding studies using quantitative methods, qualitative narrative synthesis was performed to extract predictors. Study findings that were pertinent to the current review’s scope were summarized and discussed by all three researchers (CL, PL, and KG). Researchers evaluated study design to extract barriers and facilitators of GT uptake, CT uptake, as well as individuals’ intention to invite their family for CT. Due to the limited number of quantitative studies identified and inconsistencies within study design and outcome measures, only proband intentions to invite family for CT could be extracted, and a meta-analysis could not be conducted. Quantitative data is presented in a separate section to supplement qualitative data on CT.

### Risk of Bias Assessment

Risk of bias was assessed using the Consolidated Criteria for Reporting Qualitative Research (COREQ) checklist [13], in line with systematic reviews of other reviews who used this as rating (e.g., [14–17]).

## Results

### Study Selection

Figure 1 depicts the screening and inclusion process. A total of 387 citations were identified through searches of electronic databases and 43 additional records were identified through reference mining. Full texts were further obtained for 40 citations identified, of which 15 studies (qualitative *N* = 9 and quantitative *N* = 6) met inclusion criteria.

### Study Characteristics

Studies included encompassed diverse stakeholder perspectives, including perspectives of probands, healthcare providers, and family members. Studies were classified into (1) genetic testing studies (herein referred to as GT studies), i.e., focusing on experiences and decision-making considerations affecting one’s own decision to undertake GT, and (2) cascade screening studies that focused on perspectives of probands who either conducted cascade screening invitations with family, or were deciding whether to invite family members for CT (herein referred to as CT studies).

Sample sizes varied from *N* = 243 for qualitative studies to *N* = 272,711 for quantitative studies, with a cumulative total of *N* = 272,954 participants, mostly identified as Caucasian. Studies were mainly conducted in high-income countries, including the USA (*N* = 7 studies) [18–24], the UK (*N* = 5 studies) [25–29], the Netherlands (*N* = 2 studies) [30, 31], and Australia (*N* = 1 study) [32] (see Table 1).

COREQ scores of qualitative studies ranged from 11 to 21, with a median score of 15 that is consistent with moderate reporting standards. Details of all studies can be found in Table 1.

### Results from Qualitative Studies

Thematic analysis of the qualitative studies indicated a total of 12 themes on the barriers and facilitators of GT and CT. Barriers of GT were financial concerns, suboptimal clinical care, and GT has no/low value, while facilitators of GT included family history of illness, being well informed, and GT has value. Barriers of CT were disconnect from family, emotional costs, and no value for knowing FH status, while facilitators of CT included responsibility to family, healthcare providers’ (HCPs) support for CT, and gains of CT. These are elaborated in the following sections, with quotes from studies provided where relevant.

### GT: Barriers and Facilitators

Barriers and facilitators specific to GT uptake were derived from thematic analysis. Three facilitators and barriers were identified (Table 2).

**Table 2:** Factors affecting GT uptake

Facilitators of genetic testing	Studies included	Barriers to genetic testing	Studies included
Family history of illness - Prior exposure to CHD/high cholesterol	[18, 25, 27, 28]	**Financial concerns** - Insurance - Cost of testing	[18, 31]
“Being well informed” - Information about testing and FH - Experience with other genetic testing	[18, 26, 27]	**Suboptimal clinical care** - GPs’ insufficient knowledge - Inefficient clinical process - Conflicting clinical advice - Lack of personalized follow-up	[18, 27, 31]
“GT has value” - Proactive health management with test results - Confirm genetic cause - Advance scientific research	[18, 25]	**“GT has no/low value”** - No insight on cause or actionable targets	[25, 27, 29, 32]

#### Facilitators of GT Uptake

Facilitators included family history of illness, being well informed, and perceptions on GT value.

##### Family History of Illness

Family history of adverse heart events emerged as a facilitator for genetic testing. Individuals articulated that prior exposure to CHD [18, 25] and high cholesterol [27, 28] within their family made them receptive towards testing to clarify their own FH risk. Even when individuals did not know the precise details of their family history, any awareness of adverse health events sufficed in motivating individuals to seriously consider FH GT: “The first reason is I don’t know a lot of my family history. I mean I know my mom, my dad, but I didn’t know a lot of my grandparents so some of my medical history. Some of the things that I go through might be because it’s just a genetic thing, you know” [18].

##### Being Well Informed

Having access to adequate information and resources about GT made individuals feel positively about genetic testing [26, 27]. Written information reinforced prior clinical communication, clarifying the basic concepts needed to grasp GT: “…it put in writing what the doctor had explained and, you know, for a non-medical person it’s sometimes difficult to…you think you’re following something when it’s being explained to you but then afterwards you’re a little bit confused and it’s good to have it in writing” [26].

  Prior experience with GT [18] provided information on what to expect during GT, contributing to a sense of readiness to undertake the genetic test for FH: “I don’t recall   having any questions about it or concerns because of the fact that I have already done  DNA testing previously, so it didn’t. It didn’t bother me. I mean, maybe some other people might be concerned about privacy” [18].

##### GT Has Value

Uptake of GT was driven by individuals ascribing tangible or emotional value to undergoing the process [18, 25]. Respondents noted perceived personal gains through GT, including managing health matters proactively (i.e., making good decisions to improve their health decisions in medication, lifestyle, and diet) [18]; “I think, for personal reasons, I’d like to know if there’s like a potential issue that I could avert.”

  Conclusive test results hold value to individuals, as they formally confirm genetic risk within the family, which is useful to know [25]: “Well, it’s just confirmed exactly what   you know is probably going through the family. You know, it probably is a genetic thing and, you know, if we had all been tested, fully tested, then we probably would know that this is going through the family. And it’s not going to stop at me, you know, the blood line’s going to go somewhere else … I don’t think it’s made any changes, I know that because it has went through the family the possibilities are that it was genetic anyway. So it’s not come as a surprise or anything like that, it’s something that’s there” [25].

  Other than personal gains, uptake of GT was also spurred by an identification of broader gains. Particularly, individuals recognized that their participation in GT may contribute to the advancement of scientific knowledge, which could benefit others [18]. “And it seemed to me like that’s kind of the future of where this kind of thing is going, where you can actually use someone’s DNA to maybe give them a chance at knowing what their future could be” [18].

#### Barriers of GT Uptake

Those who declined testing expressed their own reasons for not coming forward, and these perspectives converged with perspectives of probands and healthcare providers about the general challenges associated with making decisions on GT for FH. Integrating all perspectives, the following GT barriers were identified: financial concerns, suboptimal clinical care, and low/no value in testing.

##### Financial Concerns

Direct costs of GT and the impact of test results on insurance costs reduced willingness to engage in GT [18, 31]: “Who will have access to my information? Specifically, I wouldn’t want an insurance company to have access and deny me insurance because I may be a high-risk person to insure if something shows in my test” [18].

##### Suboptimal Clinical Care

Concerns about quality of clinical care discouraged GT uptake. Participants voiced doubts about (a) competence and knowledge of GT among HCPs, and (b) the onerous and inefficient post GT care structures (i.e., need for referrals [31], conflicting clinical advice [27], and absence of personalized follow-up after GT [18]).

  General practitioners (GP) reflected that they had insufficient knowledge about genetic tests and the required further assessment [31]. Additionally, GPs reported challenges pertaining to connecting patients with a wide range of specialists after GT, primarily  speaking to the difficulties to oversee additional referrals and appointments, reflecting the lack of connectedness between different institutions spanning a specific healthcare system [31].

 These concerns were also reflected by probands, who expressed that post-GT care structures were poorly integrated, hindering individuals from understanding the frequency of required clinical visits to undergo a test and interpret their test results. It was deemed  undesirable to receive test results when there would be no clear clinical follow-up to convey its significance to their own lives: “So I did all of this and it is helpful research to the state of South Carolina. But what does this mean for me? How do I navigate it and am I going to do anything with it? I mean, that’s a personal choice, but how do I get that information?” [18].

 Probands also conveyed that conflicting clinical advice was provided by various clinicians—either about the necessity of GT and/or the specific interpretation of their test results—doubting the quality of clinical care available for those receiving a FH genetic  test: A GP “more or less laughed him (son of patient with positive mutation) out of the office and said, ‘Don’t worry, you’re too young to worry about things like that.’” [27], providing no clarity as advice previously received about the need for a genetic test both for herself and her family members was contradictory.

##### GT Has No/Low Value

GT offers no additional insight about the cause of FH nor actionable targets, if individuals were already abiding by healthy lifestyle practices [25, 27, 29, 32]: “It (i.e., DNA result) still doesn’t answer the question about why you have got it; does it? They ruled that out, yeah fine. You can get on with the research, yeah. But it doesn’t answer my question: why do I have this? As I say, I think I am pretty healthy, I thought I was … When I speak to people, hardly anybody can believe that I have high cholesterol. You know, as I say I’m fairly active. I’m not overweight. I watch what I eat, but if I wasn’t on these statins my high cholesterol would be way off the scale; but why is it?” [25].

### CT: Barriers and Facilitators

CT is a dynamic process primarily involving two stakeholders: (1) probands, who initiate the disclosure of their FH results with their relatives, inviting family to confirm their own FH risk via available genetic testing services, and (2) probands’ relatives, those notified of their family risk and faced with a decision to be beneficiaries of testing. Our synthesis of CT data for FH revealed that studies delineated the perspectives of the former rather than the latter clearly, even when relatives may have been interviewed when accepting the opportunity to confirm their participation in CT. Hence, the barriers and facilitators extracted for CT focus solely on factors influencing probands conducting a family invitation (“CT invitation”).

Synthesis of data indicated five themes of facilitators of CT (responsibility to family, HCP support for CT, gains of CT, and three themes of barriers (disconnect from family, emotional costs, and no/low value for knowing FH status); see Table 3).

**Table 3:** Barriers and facilitators of CT

Facilitators of cascade testing	Studies included	Barriers to cascade testing	Studies included
Responsibility to family - Duty to inform family members - Respect for autonomy in decision-making	[26, 28, 32]	**Disconnect from family** - Emotional and geographical distance	[32]
HCPs support for CT - Direct and persuasive communication - Multi-disciplinary network - Trusting relationships	[26, 30, 31]	**Emotional costs** - Emotional stress in disclosing - Anticipated distress to family members	[18, 25, 27, 29–31]
Gains of CT - Family treatment - emotional support from family	[25, 28, 30]	**No value for knowing FH status** - FH not threatening - Labels undesirable for children	[25, 28, 30]

#### Facilitators of CT Uptake

##### Responsibility to Family

A strong sense of responsibility towards one’s family was a strong facilitator, making probands more open to initiating CT invitations [26, 28]. Individuals used familial duty to define their own responsibility in alerting their family members about FH risk, stressing that this was all they could do: “Well I talked to m’ family initially, cos I wanted them all to come down, but I couldn’t convince them all at the time. I made sure they all know, because I want them to be aware of it, ‘cos it could affect them” [28].

 Individuals had another responsibility relating to family, namely a respect for family members’ autonomy to make their own decisions, using this responsibility to set a boundary in inviting family: to inform family about the availability of testing services and not persuade them such that they intrude in family members’ decision-making about testing. These boundaries allowed probands to conclude the invitation promptly, ensuring the CT invitation remained a positive experience: “It’s not my responsibility… you can lead a horse to water… I’ve got a cousin who has not consented to screening… We know he’s got high cholesterol and doesn’t look after himself very well… I wouldn’t want that responsibility to be chasing him up and that’s why I’d rather leave it” [32].

##### HCPs Support for CT

Direct supportive communication by HCPs for CT, especially when based on a prior relationship of trust and supportive care structures (i.e., multidisciplinary teams), guided probands to stress the importance of the CT invitation [17, 21, 22]: “[Lipid consultant] was very clear that the family should take this seriously, and I’ve conveyed that to them” [26].

Risk information, when communicated by HCPs through visual and verbal aids, supported probands in communicating tailored information about FH family risk, supporting their CT invitation efforts [26]. Particularly, HCPs explanations when visually represented, furthered family members’ understanding of the need for CT [26]: “then showed me exactly, from the wee diagram, how it can work… it was the use of the arrows explaining how I could have it and then… But it was just a wee diagram that she had and it was really when you put it all together and you look at it you’re like “uh, the odds don’t look good!”.

##### Gains of CT

Advocating for CT had benefits, including getting family to seek treatment [28], and opportunities to receive emotional support from family members upon disclosure of FH proband status [30].

CT could benefit family, particularly younger members, by ensuring they receive prompt medical attention upon confirmation of a genetic cause [25, 28]: “I wanted to make sure that all the family, the kids, if they needed the treatment they got it straight away and we make sure that they take their medication and I would think that they’re probably alright and live a normal life [.] It’s because we’ve started them at a young age, it’s a habit [.] I mean my children have got it and I don’t see how it will affect their lives at all. Dietary, I mean you’ve got to give some consideration to not taking the children to MacDonald’s everyday for their meals and you know we tend to eat lots of white meat [.]” [28].

#### Barriers of CT

Barriers to CT uptake entail disconnect from family, emotional costs, and no value for knowing FH status.

##### Disconnect from Family

Probands expressed geographical and/or emotional distance within the family network as affecting their capacity to initiate a CT invitation. Particularly, probands felt out of place advocating for CT when familial ties are not strong: The reason I haven’t [contacted relatives] is because I’m not closely affiliated with them…they live overseas and I don’t really see or talk to them much” [32].

##### Emotional Costs

Individuals expressed two types of emotional costs: the emotional burden to themselves in trespassing family members’ autonomy, and the anticipated harm they may inflict on vulnerable family members when informing them about FH.

Advocating for CT was taxing as invitees were reluctant to violate the autonomy of their family members by interfering in their decision-making process about CT [18, 29–31]: “If they would smoke, I would not throw away their cigarettes either. I think you can’t interfere with that” [30].

Additionally, invitees feared that they would cause distress to family members. Consequences of disclosure of genetic risk to emotionally vulnerable family [30], or harm should inconclusive/negative DNA results be obtained by family members [25, 27] made probands reconsider their willingness to conduct a CT invitation.

 Individuals prioritized protecting emotionally vulnerable family members from further harm in knowing about their genetic risk for FH [30]. Additionally, probands own experiences and understanding of receiving negative or inconclusive test results made them worry about the potential distress if family members were to receive such results [25, 27, 31]: “If you had told me it was because of my mother, I would have been happier now, I would have thought, ‘well there is nothing I can do about that, I have inherited this and I ought to tell my daughter’ … but actually when the letter came back saying that it was negative I thought well, that just must mean that it was my fault, so I must admit [that] has made me more miserable” [27].

##### No Value for Knowing FH Status

Verifying the genetic nature of FH has no value for individuals with FH and/or their family members, as (1) FH is not considered a threatening illness [30], and (2) parents with FH viewed test results a form of “labelling,” which they deemed undesirable for their children [25, 30].

FH is perceived as not being dangerous as it alone does not affect the likelihood of CVD, making test results seem insignificant: “There are people who do have a good cholesterol [level], yet who at my age are seeing the cardiologist, so don’t bother. If I really had thought it was life threatening, I would have said: ‘Boys, watch out, don’t ignore it’” [30]. Additionally, FH labels were perceived as unfavorable for children as parents saw that their role in supporting their children to make healthy lifestyle choices from an early age as sufficient in managing FH risk: “He’s very active. He eats the diet that I eat and I just feel now he’s a child and I want him to be that with no label attached. And if he follows that regime for now, okay” [28].

### Results from Quantitative Studies

We identified *N* = 6 quantitative studies on GT or CT for FH [19–24]. Synthesis of these studies is however limited as there was significant variability in measures used and outcomes (CT action/intention and/or intention to pursue GT). Each study focused on different concepts/parameters with little consistency across the six studies with result reports not always clearly noting if measured factors were associated GT, CT, or both (Table 1). We hence only report the results that are clearly signposted for one outcome, namely intention of probands to invite family to CT (*N* = 4) [19–21, 23]. Derived themes include emotional distress avoidance [22, 23], limited opportunity to invite to CT (no contact with family or no time for family disclosure) [23], lack of knowledge [21, 23, 24], low communication efficacy [20, 23], and difficulties accessing testing services due to limited availability [24]. These themes are in line with data from qualitative studies with the exception of difficulty accessing testing services, which is reflected only in one quantitative study [24].

## Discussion

This is the first systematic review to synthesize emerging qualitative and quantitative evidence on drivers and barriers on FH-related genetic and cascade testing to inform the optimization of these services. Results indicated that GT uptake is linked to intrapersonal factors (i.e., understanding of family medical history, knowledge about FH, perceptions of testing necessity/benefits) and system-level factors (i.e., testing costs, positive results affecting insurance coverage, and quality of clinical care). CT, on the other hand, is affected by intrapersonal (no value for knowing FH status, emotional stress disclosing), interpersonal (family dynamics and responsibility, gains from family treatment), and systemic influences (HCPs support for CT).

Critical in decision-making for GT and CT is knowledge about FH and testing. Knowledge has long been considered an enabler of screening uptake for various hereditary conditions [33–35] and our synthesis indicates that both informational and experiential knowledge drive individual decision to pursue FH GT. Individuals formalize FH risk through the vicarious experiences of family members with high cholesterol or CHD, or direct exposure from undergoing genetic testing for other conditions. Knowledge on risk likely impacts genetic literacy as it is strongly associated with a willingness to test for FH.

However, as widely recognized in genetic testing literature, knowledge itself is insufficient in motivating health behaviors as individuals likely hold different attitudes towards FH and/or GT that affect their decision to test [36]. Particularly, perceptions related to benefits/value of GT (e.g., testing necessity, and gains to self, family, or society) and perceived consequences of test results (e.g., confirming genetic cause, significance of obtaining test results on lifestyle, and treatment options) impact decisions to undergo GT/CT. Individuals are more likely to attend GT, and/or to invite their family members for CT when they perceive GT as having personal (i.e., own health or their family’s), or societal value (i.e., scientific progress). Particularly, individuals are hesitant about coming forward for GT when they perceive no or low value in testing, or when they perceive GT results as capable of causing harm (e.g., unhelpful labels for children). Our findings indicate that individuals evaluate the value of genetic testing on a multidimensional scale, consolidating individual, relational, and societal considerations to arrive at a decision [37]. In light of this, risk–benefit analysis for preventative health behaviors as noted in several theoretical frameworks (Health Belief Model [38], Health Action Process [39]) has a wealth of empirical support especially in the context of GT uptake for hereditary cancers [35, 40].

Decisions related to testing are especially sensitive to interpersonal forces. Of particular salience is the role HCPs play in influencing individual decision-making regarding GT/CT. Conflicting clinical advice by HCPs and insufficient knowledge on testing among GPs may hinder uptake of GT, while direct and persuasive communication from HCPs, when provided within the safety of trust experienced by patients in pre-existing relationships and supportive clinical networks, may improve the likelihood of probands initiating CT invitations, exhibiting consistency with findings in prior cancer GT/CT work [41].

The role that HCPs play in supporting GT/CT engagement for FH cannot be understated. Healthcare systems would benefit from developing standardized resources to support HCP consultations targeting FH GT uptake. Various strategies to improve HCP’s ability to support probands conducting CT invitation have been proposed in cancer CT literature. Some examples include providing educational leaflets for patients to distribute to their relatives, writing letters of support to forward to family, and scheduling follow-up sessions with family members [26, 40, 42]. Such strategies may be implemented in FH/cardiology clinics. Additional support to HCPs may also come in the form of calibrating systemic workflow, including allocating additional personnel to support the informational and emotional needs of probands and their relatives (i.e., genetic counsellors and multi-disciplinary clinical team), and the development of standardized clinical guidelines for clinical staff to tailor communication—about FH risk and testing benefits—across diverse patient populations [43–47].

Genetic testing and cascade testing do not exist in a social vacuum. Proband decision to disclose their test results may impact family networks specific health risks for family members are not confirmed until family members obtain their own test results. Proband perception of familial ties impacts their willingness and confidence in conducting the CT invitation. Often, a lack of strong ties contributes to suboptimal communication between the proband and family during the CT invitation, emphasizing familial disconnect as a key barrier to CT uptake.

Prior literature recognizes the complexity underlying decisions associated with uncovering one’s genes through a test with the terms “genetic responsibility” or “genetic prudence,” a novel individual responsibility faced by those navigating hereditary conditions [48]. Our results indicate that conceptions of “genetic prudence” may influence probands extending CT invitations to specific family members, as individuals evaluate familial ties when extending a CT invitation. This may in part explain our finding that probands cope with the weight of this responsibility by either fully avoiding it, or by engaging with it only with clearly established limits to promote healthy interactions (i.e., simply informing family about FH and not actively persuading them to test for themselves). Overall, probands would benefit from resources explaining “genetic responsibility,” offering guidance on managing the emotional demands associated with a CT invite. Especially when familial ties are weak, perceived burdens of the CT invite can be alleviated, with the potential to improve proband engagement with more distant at-risk family members, improving uptake.

Consistent with prior literature, emotional and geographical factors and a concern for family members’ well-being heavily factor in probands’ decisions to invite relatives for CT [35, 40, 49]. Probands likely evaluate their “closeness”—merging geographical and emotional measures—when considering various family members, arriving at a calibrated decision on whether to conduct a CT invitation. As per our findings, probands’ perception of anticipated harm to family members is a strong barrier to initiating CT invitations. Consistent with prior findings in FH and cancer [25, 46], probands need to be reassured that the CT invitation can simply provide resources to support family members’ needs, overcoming probands fear of resultant harm to certain vulnerable relatives. Probands would benefit from information on the clinical structures in place to support their family members, highlighting the role of clinics in providing personalized support in helping family members make an informed decision on the test and process their results. While family dynamics are complex and likely difficult to modify, clinics may benefit from developing guidelines for probands to use when engaging in CT invitations. These guidelines should clarify to probands that the CT invitation is a proactive conversation with family members about ways to access genetic testing for FH, alongside passing along insights on the potential advantages in testing to manage FH health risks. Additionally, where appropriate, probands should be notified that they may engage HCPs to provide individualized support in tailoring family communication. Digital tools can improve proband confidence when undertaking CT invites. Widely endorsed in prior work on CT, these tools show promise in easing the emotional burdens of the process [50, 51]. While probands’ experiences on CT are well-understood, more work is needed to understand the concerns that relatives (i.e., second- and third-degree relatives) may have about receiving CT invitations from probands, and the specific resources clinics can administer to probands, ensuring CT is interpreted accurately beyond proband communication to promote its optimization [34, 52].

Our review also identified system-level barriers dissuading GT uptake. Most notably, a lack of standard workflow to oversee post-GT referral and care deters individuals from going through testing. Our data indicates that individuals may lack incentive and/or fail to understand the complex clinical pathways they must go through to qualify for the test, and what to expect after it [53]. Consistent with findings in GT interventions for other hereditary conditions, FH clinics should implement a streamlined protocol to seamlessly align HCPs from various departments (i.e., genetic counsellors, general practitioners, endocrinologists, and cardiologists) to identify and manage pre- and post-test follow-ups [54]. Some findings from cancer GT literature are also reflected in our FH-specific review, including concerns about costs of testing and genetic counselling, and the impact of positive test results on insurance coverage [35, 41, 43, 55–58]. Cost and insurance-related concerns emerge in various genetic testing realms, indicating that it needs to be urgently addressed at the national level. Clinics should be mandated to engage in transparent disclosure of the privacy policy in managing sensitive patient data from FH testing and governments should design policies to subsidize cost for low socio-economic groups and additionally administer laws against genetic discrimination from insurance providers to improve patient trust in pursuing genetic testing services.

Overall, our findings emphasize that GT and CT interventions should address different considerations: GT interventions should focus on enhancing individual perceptions about testing value and emphasize the long-term health risks of FH, while CT interventions should address the complexities of the family invitation via a wide-range of direct and illuminating resources for use among probands, their family members, and clinicians. Interventions for GT should extend beyond conventional genetic education, shaping individual perception of the value of testing, while CT interventions should equip individuals with the interpersonal skills to advocate for CT as a resource available to family members to understand and manage their own FH risk. Specialized resources focused on communication guidelines and knowledge on what to expect from testing should be developed for use across clinical settings to help probands effectively manage familial interactions, cultivating empathetic and effective risk-disclosure to promote CT engagement. Notably, interventions targeting emotional resilience have been found effective in improving risk disclosure, improving screening and diagnosis for FH [59]. The inclusion of genetic counselling within clinical care should be considered, given its ability to supplement HCP communication with at-risk relatives who have been referred to test by probands [34, 60].

To our knowledge, this is the first mixed-methods systematic review in the context of FH to synthesize evidence on barriers and facilitators of uptake of GT and CT separately. Although the number of included studies is small, the findings have important clinical implications. First, they are imperative to developing resources for both at-risk individuals and HCPs since knowledge in both stakeholder groups must be strengthened to promote testing uptake. Sufficient training of HCPs, together with educational materials for distribution to at-risk individuals and their families, is a key step in strengthening patient confidence in genetic testing. Materials for at-risk individuals should be framed to emphasize the value in testing (including personal, familial, and societal benefits), cultivating a positive perception about exercising genetic responsibility. Additionally, HCPs should evaluate individuals’ beliefs about FH and GT, and tailor communication to clarify individuals’ misconceptions about FH risk and testing. To address systemic barriers relating to clinical pathways and workflow, streamlined protocols for post-test results disclosure, next steps, and clear referrals, should be established to effectively connect HCPs from different settings, providing consistent and reliable clinical care for probands and their at-risk family members. Clear information about clinical pathways, testing procedures, and costs associated with FH care and data management policies for test results should be highlighted to at-risk individuals prior to testing in simple and clear language, with the opportunity to clarify questions during genetic counselling. Finally, public policy should take into account potential negative consequences of GT (e.g., stigmatization), and craft viable policies protecting individuals against discrimination relating to insurance coverage[47, 61], such as the United States Genetic Information Nondiscrimination Act [62].

This review has some limitations. The driving forces influencing proband decisions, to invite family members, alongside and perspectives of other stakeholders (i.e., HCPs and family members), remain under-represented in GT literature at large, as reflected in our data being skewed towards proband perspectives. Additionally, all CT data was from the perspective of probands conducting CT invitations, limiting our understanding of relatives’ experiences in receiving this communication. Since CT invitations are dynamic, our themes which reflect primarily proband experiences, may not capture the full spectrum of enablers and deterrents to CT.

Limited quotations were available in several studies in supporting their generated themes. Overall, a median COREQ score of 15 was reported across qualitative studies, reflecting a *moderate* level of reporting consistency across studies. Given the diversity of methodological orientations underlying qualitative studies, future studies should prioritize a systematic documentation of their work to ensure replicability of their findings. Conducted in English, the search limits the potential demographic cohorts, including experiences from under-represented countries, ethnic, and religious minorities. Significant differences in study design and participant demographics within quantitative data made it challenging to precisely map onto synthesized quantitative data. Overall, more empirical and statistical work in qualitative and quantitative domains is needed to clarify the impact of specific determinants on FH GT/CT testing outcomes.

## Conclusion

In conclusion, there is limited empirical research on the barriers and facilitators to FH GT/CT, affecting the timely diagnosis and overall management of cardiovascular disease prognosis. Notably, this review contributes to literature on genetic conditions, specifically cardiovascular diseases through emphasizing: (1) the pivotal role of individuals’ perception of testing value as both a barrier and facilitator to GT/CT, and (2) the significance of empowering patients through clear resources and robust clinical care structures to create a seamless pre- and post-testing experience. Community-based and clinical interventions should adopt a patient-centered care approach to improving risk-perception about FH to encourage individual decisions in GT. Innovative resources to tackle the emotional burden of family communication, alongside clinically endorsed guidelines on CT invitations, can boost proband competence in initiating CT invitations across diverse healthcare contexts.

## Supplementary Information

Below is the link to the electronic supplementary material.

PRISMA 2020 Main Checklist (DOCX 31 KB)

Search Strategy (DOCX 473 KB)

## Data Availability

Data analyzed for the review is available upon reasonable request.

## References

[1] Hu P, et al. Prevalence of familial hypercholesterolemia among the general population and patients with atherosclerotic cardiovascular disease: a systematic review and meta-analysis. Circulation. 2020;141(22):1742–59. 10.1161/CIRCULATIONAHA.119.044795. 32468833 10.1161/CIRCULATIONAHA.119.044795

[2] Reducing the global public health burden of familial hypercholesterolemia: more work ahead | Blogs | CDC. Accessed: Jul. 18, 2024. [Online]. Available: https://blogs.cdc.gov/genomics/2020/01/21/reducing-the-global/.

[3] WHG Programme, Familial hypercholesterolaemia (FH) : report of a second WHO consultation, Geneva, 4 September 1998, Art. no. WHO/HGN/FH/CONS/99.2, 1999, Accessed: Jul. 18, 2024. [Online]. Available: https://iris.who.int/handle/10665/66346.

[4] Nordestgaard BG, et al. Familial hypercholesterolaemia is underdiagnosed and undertreated in the general population: guidance for clinicians to prevent coronary heart disease : Consensus Statement of the European Atherosclerosis Society. Eur Heart J. 2013;34(45):3478–90. 10.1093/eurheartj/eht273. 23956253 10.1093/eurheartj/eht273 PMC3844152

[5] Lee S, Akioyamen LE, Aljenedil S, Rivière J-B, Ruel I, Genest J. Genetic testing for familial hypercholesterolemia: impact on diagnosis, treatment and cardiovascular risk. Eur J Prev Cardiol. 2019;26(12):1262–70. 10.1177/2047487319829746. 30755017 10.1177/2047487319829746

[6] Yu Y, et al. Association between familial hypercholesterolemia and risk of cardiovascular events and death in different cohorts: a meta-analysis of 1.1 million subjects. Front Cardiovasc Med. 2022;9:860196. 10.3389/fcvm.2022.860196. 35800161 10.3389/fcvm.2022.860196 PMC9253470

[7] Umans-Eckenhausen MA, Defesche JC, Sijbrands EJ, Scheerder RL, Kastelein JJ. Review of first 5 years of screening for familial hypercholesterolaemia in the Netherlands. Lancet Lond Engl. 2001;357(9251):165–8. 10.1016/S0140-6736(00)03587-X. 10.1016/S0140-6736(00)03587-X 11213091

[8] Ray KK, et al. Premature morbidity and mortality associated with potentially undiagnosed familial hypercholesterolemia in the general population. Am J Prev Cardiol. 2023;15:100580. 10.1016/j.ajpc.2023.100580. 37727649 10.1016/j.ajpc.2023.100580 PMC10506055

[9] Medeiros AM, Bourbon M. Genetic testing in familial hypercholesterolemia: is it for everyone? Current Atherosclerosis Reports. 2023;25(4):127–32. 10.1007/s11883-023-01091-5. 36862327 10.1007/s11883-023-01091-5 PMC10027780

[10] Schmidlen T, Hatchell K, Bristow SL, Haverfield E. eP307: the impact of proband indication for genetic testing on the uptake of cascade testing among relatives. Genet Med. 2022;24(3 Supplement):S193–4. 10.1016/j.gim.2022.01.343. 10.3389/fgene.2022.867226 PMC9243226 35783293

[11] Page MJ, et al. The PRISMA 2020 statement: an updated guideline for reporting systematic reviews. BMJ. 2021;372:n71. 10.1136/bmj.n71. 33782057 10.1136/bmj.n71 PMC8005924

[12] Braun V, Clarke V. Using thematic analysis in psychology. Qual Res Psychol. 2006;3(2):77–101. 10.1191/1478088706qp063oa.

[13] Tong A, Sainsbury P, Craig J. Consolidated Criteria for Reporting Qualitative Research (COREQ): a 32-item checklist for interviews and focus groups. Int J Qual Health Care. 2007;19(6):349–57. 10.1093/intqhc/mzm042. 17872937 10.1093/intqhc/mzm042

[14] Sumpton D, et al. Patients’ perspectives and experience of psoriasis and psoriatic arthritis: a systematic review and thematic synthesis of qualitative studies. Arthritis Care Res. 2020;72(5):711–22. 10.1002/acr.23896. 10.1002/acr.23896 30927508

[15] Kerr M, et al. Indigenous Peoples’ perspectives of living with chronic kidney disease: systematic review of qualitative studies. Kidney Int. 2022;102(4):720–7. 10.1016/j.kint.2022.05.030. 35788358 10.1016/j.kint.2022.05.030

[16] Campbell R, Ju A, King MT, Rutherford C. Perceived benefits and limitations of using patient-reported outcome measures in clinical practice with individual patients: a systematic review of qualitative studies. Qual Life Res. 2022;31(6):1597–620. 10.1007/s11136-021-03003-z. 34580822 10.1007/s11136-021-03003-z

[17] Walker RC, et al. Patient and caregiver perspectives on home hemodialysis: a systematic review. Am J Kidney Dis. 2015;65(3):451–63. 10.1053/j.ajkd.2014.10.020. 25582285 10.1053/j.ajkd.2014.10.020

[18] Allen CG, et al. Lessons learned from the pilot phase of a population-wide genomic screening program: building the base to reach a diverse cohort of 100,000 participants. J Pers Med. 2022;12(8):1228. 10.3390/jpm12081228. 36013178 10.3390/jpm12081228 PMC9410232

[19] Benson G, et al. Medication adherence, cascade screening, and lifestyle patterns among women with hypercholesterolemia: results from the WomenHeart survey. J Clin Lipidol. 2016;10(4):937–43. 10.1016/j.jacl.2016.03.012. 27578126 10.1016/j.jacl.2016.03.012

[20] Campbell M, Humanki J, Zierhut H. A novel approach to screening for familial hypercholesterolemia in a large public venue. J Community Genet. 2017;8(1):35–44. 10.1007/s12687-016-0285-1. 27889901 10.1007/s12687-016-0285-1 PMC5222759

[21] Schmidlen TJ, Bristow SL, Hatchell KE, Esplin ED, Nussbaum RL, Haverfield EV. The impact of proband indication for genetic testing on the uptake of cascade testing among relatives. Front Genet. 2022;13:867226. 10.3389/fgene.2022.867226. 35783293 10.3389/fgene.2022.867226 PMC9243226

[22] Wand H, Sturm AC, Erby L, Kindt I, Klein WMP. Genetic testing preferences and intentions in U.S. patients with clinically diagnosed familial hypercholesterolemia. Genetics. 2019. 10.1101/587923. 10.1002/jgc4.1194 PMC13147478 31769116

[23] Wurtmann E, Steinberger J, Veach PM, Khan M, Zierhut H. Risk communication in families of children with familial hypercholesterolemia: identifying motivators and barriers to cascade screening to improve diagnosis at a single medical center. J Genet Couns. 2019;28(1):50–8. 10.1007/s10897-018-0290-0. 10.1007/s10897-018-0290-0 30109451

[24] Zimmerman J, Duprez D, Veach PM, Zierhut HA. Barriers to the identification of familial hypercholesterolemia among primary care providers. J Community Genet. 2019;10(2):229–36. 10.1007/s12687-018-0383-3. 30206796 10.1007/s12687-018-0383-3 PMC6435775

[25] Jenkins N, Lawton J, Douglas M, Hallowell N. Inter-embodiment and the experience of genetic testing for familial hypercholesterolaemia. Sociol Health Illn. 2013;35(4):529–43. 10.1111/j.1467-9566.2012.01510.x. 22897600 10.1111/j.1467-9566.2012.01510.x

[26] Hallowell N, et al. Patients’ experiences and views of cascade screening for familial hypercholesterolemia (FH): a qualitative study. J Community Genet. 2011;2(4):249–57. 10.1007/s12687-011-0064-y. 22109877 10.1007/s12687-011-0064-y PMC3215780

[27] Silva L, Condon L, Qureshi N, Dutton B, Weng S, Kai J. Introducing genetic testing with case finding for familial hypercholesterolaemia in primary care: qualitative study of patient and health professional experience. Br J Gen Pract. 2022;72(720):e519–27. 10.3399/BJGP.2021.0558. 35697509 10.3399/BJGP.2021.0558 PMC9208733

[28] Weiner K. Exploring genetic responsibility for the self, family and kin in the case of hereditary raised cholesterol. Soc Sci Med. 2011;72(11):1760–7. 10.1016/j.socscimed.2010.03.053. 20627500 10.1016/j.socscimed.2010.03.053

[29] Weiner K, Durrington PN. Patients’ understandings and experiences of familial hypercholesterolemia. Public Health Genomics. 2008;11(5):273–82. 10.1159/000121398. 10.1159/000121398 18493125

[30] Van Den Nieuwenhoff HWP, Mesters I, Gielen C, De Vries NK. Family communication regarding inherited high cholesterol: why and how do patients disclose genetic risk? Soc Sci Med. 2007;65(5):1025–37. 10.1016/j.socscimed.2007.04.008. 17507128 10.1016/j.socscimed.2007.04.008

[31] van El CG, Baccolini V, Piko P, Cornel MC. Stakeholder views on active cascade screening for familial hypercholesterolemia. Healthc. Basel Switz. 2018;6(3). 10.3390/healthcare6030108. 10.3390/healthcare6030108 PMC6163326 30200297

[32] Hardcastle S, et al. Patients’ perceptions and experiences of familial hypercholesterolemia, cascade genetic screening and treatment. Int J Behav Med. 2015;22(1):92–100. 10.1007/s12529-014-9402-x. 24585182 10.1007/s12529-014-9402-x

[33] Faradisa E, Ardiana H, Priyantini D, Fauziah A, Susanti I. A systematic review of the factors associated with cervical cancer screening uptake among women in low and middle-income countries. J Ners. 2020;15(1Sp):113–9. 10.20473/jn.v15i1Sp.18991.

[34] Grutters LA, Christiaans I. Cascade genetic counseling and testing in hereditary syndromes: inherited cardiovascular disease as a model: a narrative review. Fam Cancer. 2024;23(2):155–64. 10.1007/s10689-023-00356-x. 38184510 10.1007/s10689-023-00356-x PMC11153290

[35] Willis AM, Smith SK, Meiser B, Ballinger ML, Thomas DM, Young M-A. Sociodemographic, psychosocial and clinical factors associated with uptake of genetic counselling for hereditary cancer: a systematic review. Clin Genet. 2017;92(2):121–33. 10.1111/cge.12868. 27643459 10.1111/cge.12868

[36] Hann KEJ, et al. Awareness, knowledge, perceptions, and attitudes towards genetic testing for cancer risk among ethnic minority groups: a systematic review. BMC Public Health. 2017;17(1):503. 10.1186/s12889-017-4375-8. 28545429 10.1186/s12889-017-4375-8 PMC5445407

[37] Sivell S, et al. How risk is perceived, constructed and interpreted by clients in clinical genetics, and the effects on decision making: systematic review. J Genet Couns. 2008;17(1):30–63. 10.1007/s10897-007-9132-1. 17968638 10.1007/s10897-007-9132-1

[38] Elkefi S, Choudhury A, Strachna O, Asan O. Impact of health perception and knowledge on genetic testing decisions using the health belief model. JCO Clin Cancer Inform. 2022;6:e2100117. 10.1200/CCI.21.00117. 34990211 10.1200/CCI.21.00117 PMC9848547

[39] Schwarzer R, Luszczynska A. How to overcome health-compromising behaviors: the health action process approach. Eur Psychol. 2008;13(2):141–51. 10.1027/1016-9040.13.2.141.

[40] Afaya A, et al. Psychosocial barriers and facilitators for cascade genetic testing in hereditary breast and ovarian cancer: a scoping review. Fam Cancer. 2024;23(2):121–32. 10.1007/s10689-024-00379-y. 38662264 10.1007/s10689-024-00379-y

[41] Duenas DM, et al. Motivations and concerns of patients considering participation in an implementation study of a hereditary cancer risk assessment program in diverse primary care settings. Genet Med. 2022;24(3):610–21. 10.1016/j.gim.2021.11.017. 34906471 10.1016/j.gim.2021.11.017 PMC8939763

[42] Campbell-Salome G, et al. Developing and optimizing innovative tools to address familial hypercholesterolemia underdiagnosis: identification methods, patient activation, and cascade testing for familial hypercholesterolemia. Circ Genomic Precis Med. 2021;14(1):E003120. 10.1161/CIRCGEN.120.003120. 10.1161/CIRCGEN.120.003120 PMC7892261 33480803

[43] Kanga-Parabia A, Gaff C, Flander L, Jenkins M, Keogh LA. Discussions about predictive genetic testing for Lynch syndrome: the role of health professionals and families in decisions to decline. Fam Cancer. 2018;17(4):547–55. 10.1007/s10689-018-0078-2. 29464398 10.1007/s10689-018-0078-2 PMC6102092

[44] Farwati M, Kumbamu A, Kochan D, Kullo I. A patient decision aid for familial hypercholesterolemia based on patient and provider feedback. J Am Coll Cardiol. 2018;71(11 Supplement):1. 10.1016/S0735-1097/2818/2933194-2.29301615

[45] Nelson HD, Pappas M, Cantor A, Haney E, Holmes R. Risk assessment, genetic counseling, and genetic testing for *BRCA-* related cancer in women: updated evidence report and systematic review for the US Preventive Services Task Force. JAMA. 2019;322(7):666. 10.1001/jama.2019.8430. 31429902 10.1001/jama.2019.8430

[46] Srinivasan S, Won NY, Dotson WD, Wright ST, Roberts MC. Barriers and facilitators for cascade testing in genetic conditions: a systematic review. Eur J Hum Genet. 2020;28(12):1631–44. 10.1038/s41431-020-00725-5. 32948847 10.1038/s41431-020-00725-5 PMC7784694

[47] Hendricks-Sturrup RM, Lu CY. Understanding implementation challenges to genetic testing for familial hypercholesterolemia in the United States. J Pers Med. 2019;9(1):9. 10.3390/jpm9010009. 30717118 10.3390/jpm9010009 PMC6463173

[48] Muir LA, George PM, Whitehead L. Using the experiences of people with familial hypercholesterolaemia to help reduce the risk of cardiovascular disease: a qualitative systematic review. J Adv Nurs John Wiley Sons Inc. 2012;68(9):1920–32. 10.1111/j.1365-2648.2012.05957.x. 10.1111/j.1365-2648.2012.05957.x 22348692

[49] Wiseman M, Dancyger C, Michie S. Communicating genetic risk information within families: a review. Fam Cancer. 2010;9(4):691–703. 10.1007/s10689-010-9380-3. 20852947 10.1007/s10689-010-9380-3

[50] Sarki M, et al. Intention to inform relatives, rates of cascade testing, and preference for patient-mediated communication in families concerned with hereditary breast and ovarian cancer and lynch syndrome: the Swiss CASCADE cohort. Cancers. 2022;14(7):1636. 10.3390/cancers14071636. 35406409 10.3390/cancers14071636 PMC8997156

[51] Bangash H, Makkawy A, Gundelach JH, Miller AA, Jacobson KA, Kullo IJ. Web-based tool (FH Family Share) to increase uptake of cascade testing for familial hypercholesterolemia: development and evaluation. JMIR Hum Factors. 2022;9(1):e32568. 10.2196/32568. 35166678 10.2196/32568 PMC8889478

[52] Roberts MC, et al. Delivery of cascade screening for hereditary conditions: a scoping review of the literature. Health Aff (Millwood). 2018;37(5):801–8. 10.1377/hlthaff.2017.1630. 29733730 10.1377/hlthaff.2017.1630 PMC11022644

[53] Vogel RI, Niendorf K, Lee H, Petzel S, Lee HY, Geller MA. A qualitative study of barriers to genetic counseling and potential for mobile technology education among women with ovarian cancer. Hered Cancer Clin Pract. 2018;16(1):13. 10.1186/s13053-018-0095-z. 29997716 10.1186/s13053-018-0095-z PMC6031189

[54] Stock E, Walsh-Bailey C, Tuda D, Tabak R, Baumann A, Hagemann A. A mixed methods analysis of patient- and provider-perceived barriers and facilitators to cascade genetic testing for hereditary breast, ovarian, endometrial and colorectal cancer (2153). Gynecol Oncol. 2023;176:S228–9. 10.1016/j.ygyno.2023.06.277.

[55] Valencia OM, Samuel SE, Viscusi RK, Riall TS, Neumayer LA, Aziz H. The role of genetic testing in patients with breast cancer: a review. JAMA Surg. 2017;152(6):589. 10.1001/jamasurg.2017.0552. 28423155 10.1001/jamasurg.2017.0552

[56] Kne A, et al. Why is cancer genetic counseling underutilized by women identified as at risk for hereditary breast cancer? Patient perceptions of barriers following a referral letter. J Genet Couns. 2017;26(4):697–715. 10.1007/s10897-016-0040-0. 27826805 10.1007/s10897-016-0040-0

[57] Glenn BA, Chawla N, Bastani R. Barriers to genetic testing for breast cancer risk among ethnic minority women: an exploratory study. Ethn Dis. 2012;22(3):267–73. 22870568

[58] Keogh LA, Niven H, Rutstein A, Flander L, Gaff C, Jenkins M. Choosing not to undergo predictive genetic testing for hereditary colorectal cancer syndromes: expanding our understanding of decliners and declining. J Behav Med. 2017;40(4). 10.1007/s10865-016-9820-0. 10.1007/s10865-016-9820-0 PMC6057776 28197815

[59] Polanski A, Wolin E, Kocher M, Zierhut H. A scoping review of interventions increasing screening and diagnosis of familial hypercholesterolemia. Genet Med. 2022;24(9):1791–802. 10.1016/j.gim.2022.05.012. 35713652 10.1016/j.gim.2022.05.012

[60] Tricou EP, Morgan KM, Betts M, Sturm AC. Genetic testing for familial hypercholesterolemia in clinical practice. Curr Atheroscler Rep. 2023;25(5):197–208. 10.1007/s11883-023-01094-2. 37060538 10.1007/s11883-023-01094-2

[61] Taket A, McKay FH. Health equity and human rights,” in *Health Equity, Social Justice and Human Rights*, 2nd ed., Routledge. 2020.

[62] Genetic information | HHS.gov. Accessed: Oct. 18, 2024. [Online]. Available: https://www.hhs.gov/hipaa/for-professionals/special-topics/genetic-information/index.html.

