# Supplementary material for: Facilitators and Barriers to Uptake of Genetic and Cascade Testing in Familial Hypercholesterolemia: a Systematic Review
Source: Int J Behav Med. 2025 Apr 8;33(1):69–82. doi: 10.1007/s12529-025-10357-y (PMC12935743; doi:10.1007/s12529-025-10357-y)

Cochrane Search:

Search Name:

Date Run: 27/10/2023 08:31:54

Comment:

ID Search Hits

#1 MeSH descriptor: [Hyperlipoproteinemia Type II] this term only 601

#2 ("Familial Combined" NEXT Hyperlipoproteinemia? or "Hyper-Low Density" NEXT Lipoproteinemia? or Hyper-Low-Density-Lipoproteinemia? or Hyper-beta-Lipoproteinemia? or Hyperbetalipoproteinemia or "Hyperlipoproteinemia Type" NEXT 2? or "Hyperlipoproteinemia Type" NEXT II? or "LDL Receptor" NEXT Disorder? or familial NEXT hypercholesterolemia? or "autosomal dominant" NEXT hypercholesterolemia? or essential NEXT hypercholesterolemia? or "familial hypercholesterolemic xanthomatosis" OR "familial hypercholesterolemic xanthomatoses" or "hyper beta" NEXT lipoproteinemia? or hyper-beta-lipoproteinemia? or "familial defective apolipoprotein b 100" or "familial ligand defective apolipoprotein b 100"):ti,ab,kw (Word variations have been searched) 1252

#3 #1 OR #2 1252

#4 MeSH descriptor: [Genetic Testing] this term only 549

#5 ((genetic NEAR/2 testing) or "Genetic Predisposition Testing" or "Genetic Screening"):ti,ab,kw (Word variations have been searched) 2177

#6 #4 OR #5 2177

#7 (barrier? or facilitator? or perception? or challenge? or uptake or understanding? or experience? or preference? or intention?):ti,ab,kw (Word variations have been searched) 420875

#8 #3 AND #6 AND #7 13

CINAHL Search:


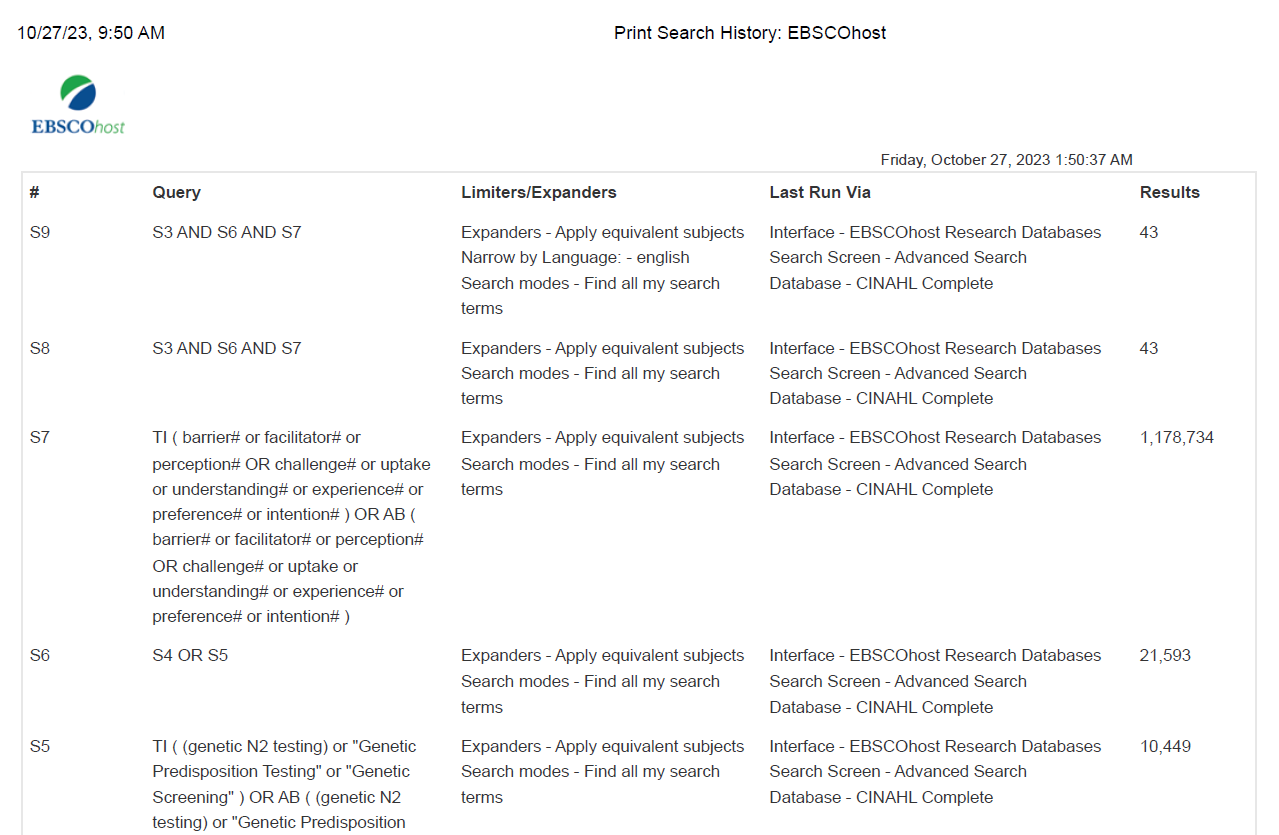

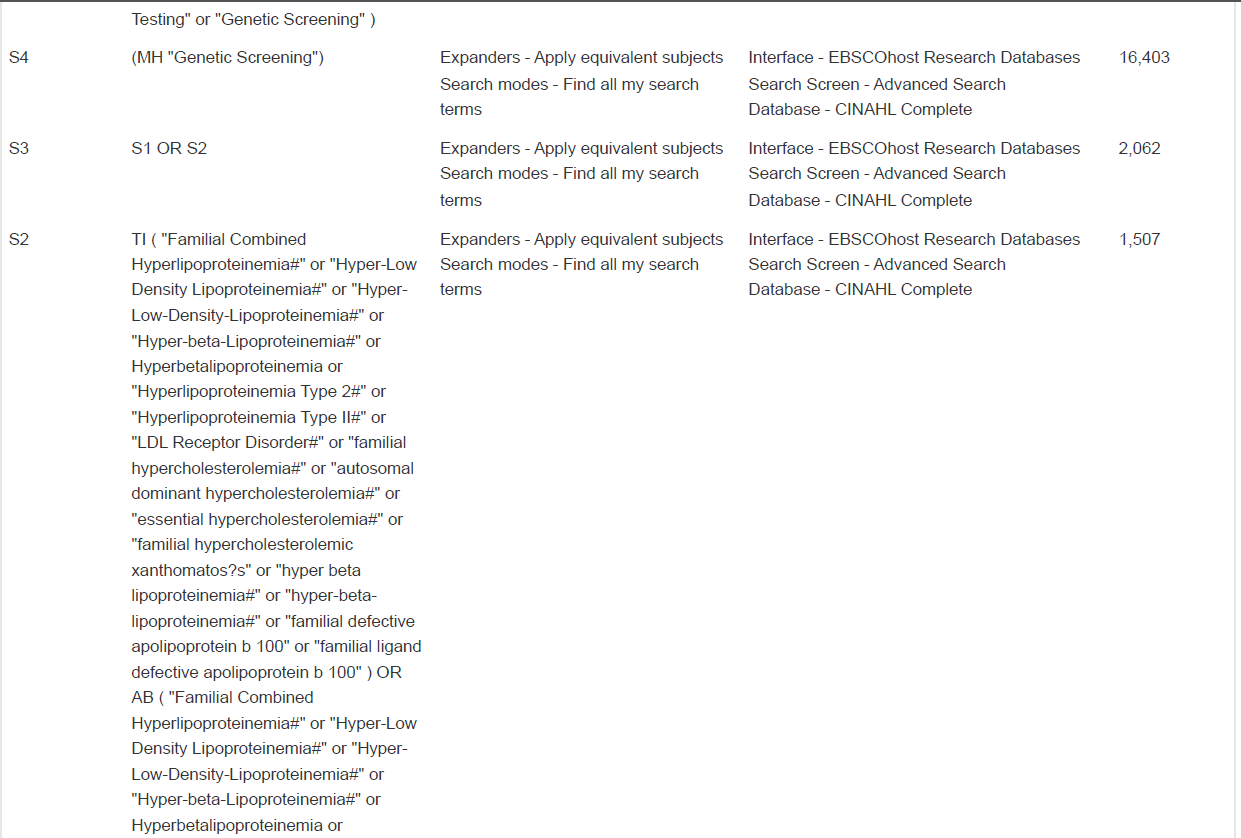

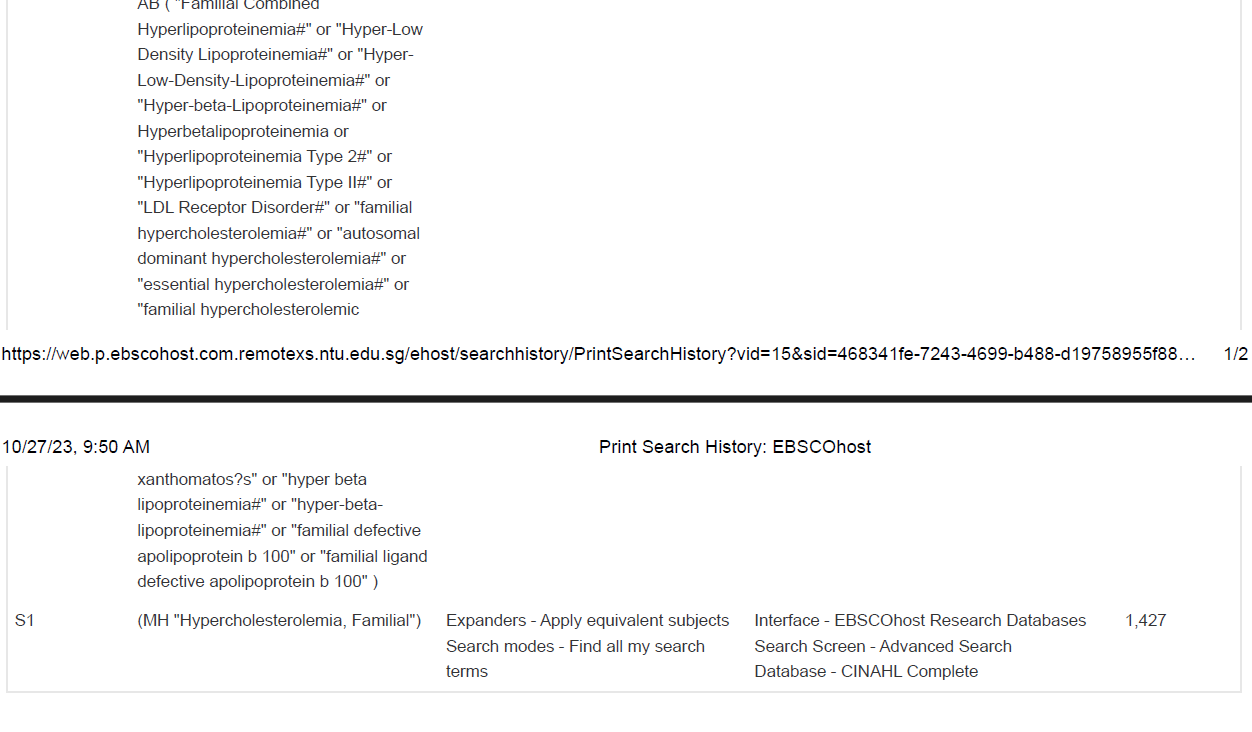


Embase (Ovid) Search:

**Database: Embase <1974 to 2023 October 25>**
**Search Strategy:**
**1**  familial hypercholesterolemia/ (12425)
**2**  ("Familial Combined Hyperlipoproteinemia?" or "Hyper-Low Density Lipoproteinemia?" or "Hyper-Low-Density-Lipoproteinemia?" or "Hyper-beta-Lipoproteinemia?" or Hyperbetalipoproteinemia or "Hyperlipoproteinemia Type 2?" or "Hyperlipoproteinemia Type II?" or "LDL Receptor Disorder?" or "familial hypercholesterolemia?" or "autosomal dominant hypercholesterolemia?" or "essential hypercholesterolemia?" or "familial hypercholesterolemic xanthomatos#s" or "hyper beta lipoproteinemia?" or "hyper-beta-lipoproteinemia?" or "familial defective apolipoprotein b 100" or "familial ligand defective apolipoprotein b 100").ab,ti. (9951)
**3**  1 or 2 (14916)
**4**  genetic screening/ (117740)
**5**  ((genetic adj2 testing) or "Genetic Predisposition Testing" or "Genetic Screening").ab,ti. (64873)
**6**  4 or 5 (136105)
**7**  (barrier? or facilitator? or perception? or challenge? or uptake or understanding? or experience? or preference? or intention?).ab,ti. (5468690)
**8**  3 and 6 and 7 (262)
**9**  limit 8 to english language (256)

Medline (Ovid) Search:

**Database: Ovid MEDLINE(R) and Epub Ahead of Print, In-Process, In-Data-Review & Other Non-Indexed Citations, Daily and Versions <1946 to October 25, 2023>**
**Search Strategy:**
**1**  Hyperlipoproteinemia Type II/ (7707)
**2**  ("Familial Combined Hyperlipoproteinemia?" or "Hyper-Low Density Lipoproteinemia?" or "Hyper-Low-Density-Lipoproteinemia?" or "Hyper-beta-Lipoproteinemia?" or Hyperbetalipoproteinemia or "Hyperlipoproteinemia Type 2?" or "Hyperlipoproteinemia Type II?" or "LDL Receptor Disorder?" or "familial hypercholesterolemia?" or "autosomal dominant hypercholesterolemia?" or "essential hypercholesterolemia?" or "familial hypercholesterolemic xanthomatos#s" or "hyper beta lipoproteinemia?" or "hyper-beta-lipoproteinemia?" or "familial defective apolipoprotein b 100" or "familial ligand defective apolipoprotein b 100").ab,ti. (6913)
**3**  1 or 2 (10292)
**4**  Genetic Testing/ (45175)
**5**  ((genetic adj2 testing) or "Genetic Predisposition Testing" or "Genetic Screening").ab,ti. (37993)
**6**  4 or 5 (69619)
**7**  (barrier? or facilitator? or perception? or challenge? or uptake or understanding? or experience? or preference? or intention?).ab,ti. (4327212)
**8**  3 and 6 and 7 (122)
**9**  limit 8 to english language (119)

PubMed Search:

| Search number | Query | Sort By | Filters | Search Details | Results |
| --- | --- | --- | --- | --- | --- |
| 9 | #3 AND #6 AND #7 | | English | (("hyperlipoproteinemia type ii"[MeSH Terms:noexp] OR ("familial combined hyperlipoproteinemia*"[Title/Abstract] OR "hyper low density lipoproteinemia*"[Title/Abstract] OR "hyper low density lipoproteinemia*"[Title/Abstract] OR "hyper beta lipoproteinemia*"[Title/Abstract] OR "Hyperbetalipoproteinemia"[Title/Abstract] OR "hyperlipoproteinemia type 2*"[Title/Abstract] OR "hyperlipoproteinemia type ii*"[Title/Abstract] OR "ldl receptor disorder*"[Title/Abstract] OR "familial hypercholesterolemia*"[Title/Abstract] OR "autosomal dominant hypercholesterolemia*"[Title/Abstract] OR "essential hypercholesterolemia*"[Title/Abstract] OR "familial hypercholesterolemic xanthomatosis"[Title/Abstract] OR "hyper beta lipoproteinemia*"[Title/Abstract] OR "hyper beta lipoproteinemia*"[Title/Abstract] OR "familial defective apolipoprotein b 100"[Title/Abstract] OR "familial ligand defective apolipoprotein b 100"[Title/Abstract])) AND ("genetic testing"[MeSH Terms:noexp] OR ("Genetic Predisposition Testing"[Title/Abstract] OR "Genetic Screening"[Title/Abstract] OR "genetic testing"[Title/Abstract:~2])) AND ("barrier*"[Title/Abstract] OR "facilitator*"[Title/Abstract] OR "perception*"[Title/Abstract] OR "challenge*"[Title/Abstract] OR "uptake"[Title/Abstract] OR "understanding*"[Title/Abstract] OR "experience*"[Title/Abstract] OR "preference*"[Title/Abstract] OR "intention*"[Title/Abstract])) AND (english[Filter]) | 126 |
| 8 | #3 AND #6 AND #7 | |  | ("hyperlipoproteinemia type ii"[MeSH Terms:noexp] OR ("familial combined hyperlipoproteinemia*"[Title/Abstract] OR "hyper low density lipoproteinemia*"[Title/Abstract] OR "hyper low density lipoproteinemia*"[Title/Abstract] OR "hyper beta lipoproteinemia*"[Title/Abstract] OR "Hyperbetalipoproteinemia"[Title/Abstract] OR "hyperlipoproteinemia type 2*"[Title/Abstract] OR "hyperlipoproteinemia type ii*"[Title/Abstract] OR "ldl receptor disorder*"[Title/Abstract] OR "familial hypercholesterolemia*"[Title/Abstract] OR "autosomal dominant hypercholesterolemia*"[Title/Abstract] OR "essential hypercholesterolemia*"[Title/Abstract] OR "familial hypercholesterolemic xanthomatosis"[Title/Abstract] OR "hyper beta lipoproteinemia*"[Title/Abstract] OR "hyper beta lipoproteinemia*"[Title/Abstract] OR "familial defective apolipoprotein b 100"[Title/Abstract] OR "familial ligand defective apolipoprotein b 100"[Title/Abstract])) AND ("genetic testing"[MeSH Terms:noexp] OR ("Genetic Predisposition Testing"[Title/Abstract] OR "Genetic Screening"[Title/Abstract] OR "genetic testing"[Title/Abstract:~2])) AND ("barrier*"[Title/Abstract] OR "facilitator*"[Title/Abstract] OR "perception*"[Title/Abstract] OR "challenge*"[Title/Abstract] OR "uptake"[Title/Abstract] OR "understanding*"[Title/Abstract] OR "experience*"[Title/Abstract] OR "preference*"[Title/Abstract] OR "intention*"[Title/Abstract]) | 129 |
| 7 | barrier*[Title/Abstract] OR facilitator*[Title/Abstract] OR perception*[Title/Abstract] OR challenge*[Title/Abstract] OR uptake[Title/Abstract] OR understanding*[Title/Abstract] OR experience*[Title/Abstract] OR preference*[Title/Abstract] OR intention*[Title/Abstract] | | | "barrier*"[Title/Abstract] OR "facilitator*"[Title/Abstract] OR "perception*"[Title/Abstract] OR "challenge*"[Title/Abstract] OR "uptake"[Title/Abstract] OR "understanding*"[Title/Abstract] OR "experience*"[Title/Abstract] OR "preference*"[Title/Abstract] OR "intention*"[Title/Abstract] | 4,371,303 |
| 6 | #4 OR #5 |  |  | "genetic testing"[MeSH Terms:noexp] OR "Genetic Predisposition Testing"[Title/Abstract] OR "Genetic Screening"[Title/Abstract] OR "genetic testing"[Title/Abstract:~2] | 72,206 |
| 5 | "Genetic Predisposition Testing"[Title/Abstract] OR "Genetic Screening"[Title/Abstract] OR "genetic testing"[Title/Abstract:~2] | | | "Genetic Predisposition Testing"[Title/Abstract] OR "Genetic Screening"[Title/Abstract] OR "genetic testing"[Title/Abstract:~2] | 41,351 |
| 4 | "genetic testing"[MeSH:NoExp] | | | "genetic testing"[MeSH Terms:noexp] | 45,160 |
| 3 | #1 OR #2 |  |  | "hyperlipoproteinemia type ii"[MeSH Terms:noexp] OR "familial combined hyperlipoproteinemia*"[Title/Abstract] OR "hyper low density lipoproteinemia*"[Title/Abstract] OR "hyper low density lipoproteinemia*"[Title/Abstract] OR "hyper beta lipoproteinemia*"[Title/Abstract] OR "Hyperbetalipoproteinemia"[Title/Abstract] OR "hyperlipoproteinemia type 2*"[Title/Abstract] OR "hyperlipoproteinemia type ii*"[Title/Abstract] OR "ldl receptor disorder*"[Title/Abstract] OR "familial hypercholesterolemia*"[Title/Abstract] OR "autosomal dominant hypercholesterolemia*"[Title/Abstract] OR "essential hypercholesterolemia*"[Title/Abstract] OR "familial hypercholesterolemic xanthomatosis"[Title/Abstract] OR "hyper beta lipoproteinemia*"[Title/Abstract] OR "hyper beta lipoproteinemia*"[Title/Abstract] OR "familial defective apolipoprotein b 100"[Title/Abstract] OR "familial ligand defective apolipoprotein b 100"[Title/Abstract] | 10,421 |
| 2 | "Familial Combined Hyperlipoproteinemia*"[Title/Abstract] OR "Hyper-Low Density Lipoproteinemia*"[Title/Abstract] OR "Hyper-Low-Density-Lipoproteinemia*"[Title/Abstract] OR "Hyper-beta-Lipoproteinemia*"[Title/Abstract] OR Hyperbetalipoproteinemia[Title/Abstract] OR "Hyperlipoproteinemia Type 2*"[Title/Abstract] OR "Hyperlipoproteinemia Type II*"[Title/Abstract] OR "LDL Receptor Disorder*"[Title/Abstract] OR "familial hypercholesterolemia*"[Title/Abstract] OR "autosomal dominant hypercholesterolemia*"[Title/Abstract] OR "essential hypercholesterolemia*"[Title/Abstract] OR "familial hypercholesterolemic xanthomatosis"[Title/Abstract] OR "familial hypercholesterolemic xanthomatoses"[Title/Abstract] OR "hyper beta lipoproteinemia*"[Title/Abstract] OR "hyper-beta-lipoproteinemia*"[Title/Abstract] OR "familial defective apolipoprotein b 100"[Title/Abstract] OR "familial ligand defective apolipoprotein b 100"[Title/Abstract] | | | "familial combined hyperlipoproteinemia*"[Title/Abstract] OR "hyper low density lipoproteinemia*"[Title/Abstract] OR "hyper low density lipoproteinemia*"[Title/Abstract] OR "hyper beta lipoproteinemia*"[Title/Abstract] OR "Hyperbetalipoproteinemia"[Title/Abstract] OR "hyperlipoproteinemia type 2*"[Title/Abstract] OR "hyperlipoproteinemia type ii*"[Title/Abstract] OR "ldl receptor disorder*"[Title/Abstract] OR "familial hypercholesterolemia*"[Title/Abstract] OR "autosomal dominant hypercholesterolemia*"[Title/Abstract] OR "essential hypercholesterolemia*"[Title/Abstract] OR "familial hypercholesterolemic xanthomatosis"[Title/Abstract] OR "hyper beta lipoproteinemia*"[Title/Abstract] OR "hyper beta lipoproteinemia*"[Title/Abstract] OR "familial defective apolipoprotein b 100"[Title/Abstract] OR "familial ligand defective apolipoprotein b 100"[Title/Abstract] | 7,079 |
| 1 | "hyperlipoproteinemia type ii"[MeSH:NoExp] | | | "hyperlipoproteinemia type ii"[MeSH Terms:noexp] | 7,708 |

Scopus Search:

( TITLE-ABS ( "Familial Combined Hyperlipoproteinemia*" OR "Hyper-Low Density Lipoproteinemia*" OR "Hyper-Low-Density-Lipoproteinemia*" OR "Hyper-beta-Lipoproteinemia*" OR hyperbetalipoproteinemia OR "Hyperlipoproteinemia Type 2*" OR "Hyperlipoproteinemia Type II*" OR "LDL Receptor Disorder*" OR "familial hypercholesterolemia*" OR "autosomal dominant hypercholesterolemia*" OR "essential hypercholesterolemia*" OR "familial hypercholesterolemic xanthomatosis" OR "familial hypercholesterolemic xanthomatoses" OR "hyper beta lipoproteinemia*" OR "hyper-beta-lipoproteinemia*" OR "familial defective apolipoprotein b 100" OR "familial ligand defective apolipoprotein b 100" ) ) AND ( TITLE-ABS ( ( genetic W/2 testing ) OR "Genetic Predisposition Testing" OR "Genetic Screening" ) ) AND ( TITLE-ABS ( barrier* OR facilitator* OR perception* OR challenge* OR uptake OR understanding* OR experience* OR preference* OR intention* ) ) AND ( LIMIT-TO ( LANGUAGE , "English" ) )

WoS Search:


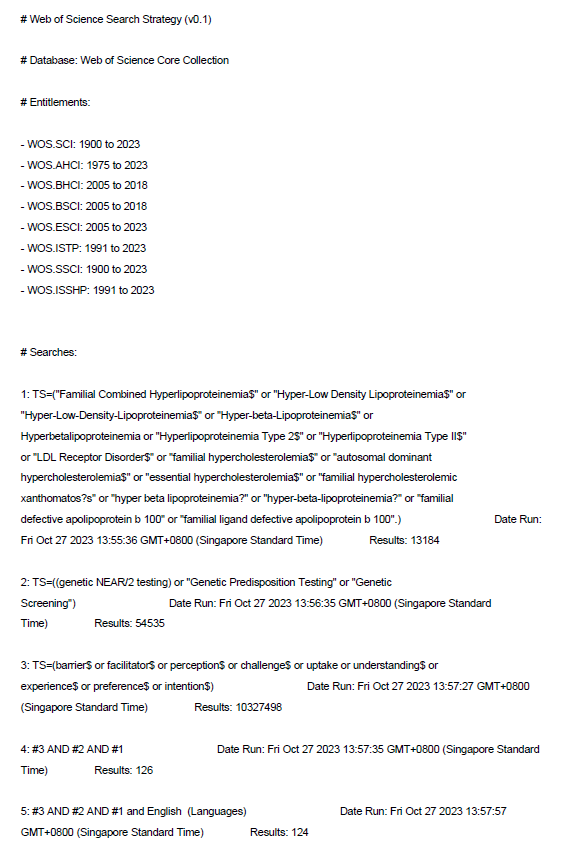

Supplement: Supplementary file 2 — Search Strategy (DOCX 473 KB) [file 12529_2025_10357_MOESM2_ESM.docx]
